# Physical activity levels in three Brazilian birth cohorts as assessed with raw triaxial wrist accelerometry

**DOI:** 10.1093/ije/dyu203

**Published:** 2014-10-30

**Authors:** Inácio CM da Silva, Vincent T van Hees, Virgílio V Ramires, Alan G Knuth, Renata M Bielemann, Ulf Ekelund, Soren Brage, Pedro C Hallal

**Affiliations:** ^1^Postgraduate Program in Epidemiology, Federal University of Pelotas, Pelotas, Brazil, ^2^Medical Research Council Epidemiology Unit, University of Cambridge, Cambridge, UK, ^3^Institute of Cellular Medicine, Newcastle University, Newcastle upon Tyne, UK, ^4^Postgraduate Program in Public Health, Federal University of Rio Grande, Rio Grande, Brazil and ^5^Department of Sport Medicine, Norwegian School of Sport Sciences, Oslo, Norway

**Keywords:** Activity monitor, cohort studies, motor activity, movement

## Abstract

**Background:** Data on objectively measured physical activity are lacking in low- and middle-income countries. The aim of this study was to describe objectively measured overall physical activity and time spent in moderate-to-vigorous physical activity (MVPA) in individuals from the Pelotas (Brazil) birth cohorts, according to weight status, socioeconomic status (SES) and sex.

**Methods:** All children born in 1982, 1993 and 2004 in hospitals in the city of Pelotas, Brazil, constitute the sampling frame; of these 99% agreed to participate. The most recent follow-ups were conducted between 2010 and 2013. In total, 8974 individuals provided valid data derived from raw triaxial wrist accelerometry. The average acceleration is presented in milli-*g* (1 m*g* = 0.001*g*), and time (min/d) spent in MVPA (>100 m*g*) is presented in 5- and 10-min bouts.

**Results:** Mean acceleration in the 1982 (mean age 30.2 years), 1993 (mean age 18.4 years) and 2004 (mean age 6.7 years) cohorts was 35 m*g*, 39 m*g* and 60 m*g*, respectively. Time spent in MVPA was 26 [95% confidence interval (CI) 25; 27], 43 (95% CI 42; 44) and 45 (95% CI 43; 46) min/d in the three cohorts, respectively, using 10-min bouts. Mean MVPA was on average 42% higher when using 5-min bouts. Males were more active than females and physical activity was inversely associated with age of the cohort and SES. Normal-weight individuals were more active than underweight, overweight and obese participants.

**Conclusions:** Overall physical activity and time spent in MVPA differed by cohort (age), sex, weight status and SES. Higher levels of activity in low SES groups may be explained by incidental physical activity.

**Key Messages**

- Objectively-measured physical activity was assessed in almost 9000 children, adolescents and young adults belonging to the Pelotas birth cohorts in Brazil.
- The mean time per day spent in moderate-to-vigorous intensity physical activity (10-min bouts) was 45 min in those aged 7 years, 43 min in those aged 18 years and 26 min in those aged 30 years.
- Males were more active than females and physical activity was lower at higher levels of SES. Normal-weight individuals were more active than underweight, overweight and obese participants.

## Introduction

Despite compelling evidence for a causal association between physical inactivity and various health outcomes,^1^ low levels of physical activity are still observed worldwide.^2^ In addition, some uncertainty remains on estimating population levels of physical activity, primarily due to difficulties in accurately assessing physical activity in surveillance systems. From a public health perspective, it is important to ascertain the level of this behaviour in the population and continuously monitor physical activity to assess temporal trends.^2^^,^^3^

With few exceptions, most large-scale population-based surveys rely on self-report methods.^2^ Data on population levels of objectively measured physical activity by accelerometry in adults and the elderly are only available from a few high-income countries. The mean time spent in moderate-to-vigorous physical activity (MVPA) was estimated at 35.5 minutes per day, using data from four countries.^2^ Available data on objectively measured physical activity suggests higher levels of time spent in MVPA in children and adolescents (<18 years) compared with adults, equating to an average of more than 60 minutes per day using data from 10 countries.^2^

Two previous studies assessing physical activity by accelerometry conducted in Pelotas, Brazil, suggested that 4–11-year-olds accumulated approximately 78 minutes per day in MVPA^4^ whereas 13-year-old children from the 1993 Pelotas cohort spent approximately 53 minutes per day at this intensity level.^5^ However, the sample size of these studies was relatively small.

Therefore, the aim of the present study was to describe objectively measured overall physical activity and time spent in MVPA in children [aged 7 years (y)], adolescents (18 y), and adults (30 y) belonging to the three Pelotas birth cohorts. We also explored differences in physical activity by sex, socioeconomic status (SES) and weight status.

## Methods

### Study design

The current study includes participants from three Pelotas birth cohorts in whom physical activity was assessed objectively between 2010 and 2013. Pelotas is a city in southern Brazil with around 320 000 inhabitants. As in most Brazilian cities, socioeconomic inequalities are marked in Pelotas (income Gini-Index: 0.42). The city’s economy is based on education and commerce. In 1982, 1993 and 2004, all children born in hospitals in the city were eligible to participate in the study (less than 1% of the deliveries were at home or other places). Refusals accounted for less than 1% of invited participants in each cohort. The three birth cohorts have been followed up at different time points thereafter. Further details about the methodology of each birth cohort are available elsewhere.^6–8^ The study and its protocols were approved by the School of Medicine Ethics Committee of the Federal University of Pelotas. All participants or their legal representatives voluntarily signed a consent letter prior to participating in the study.

The most recent follow-up visit of each cohort took place when those born in 1982 were approximately 30 years of age, those born in 1993 were 18 years of age and those born in 2004 were 7 years of age. Unlike previous data collections which were carried out at the participants’ homes, and where physical activity was assessed predominately by self- or parental report,^9^ all measurements during the most recent follow-up visits were performed at the research clinic. Following anthropometric, clinical and biochemical measurements, participants were invited and instructed to wear an accelerometer (GENEActiv; ActivInsights, Kimbolton, UK), on their non-dominant wrist. Follow-up rates were 68% in the 1982 cohort, 81% in the 1993 cohort and 90% in the 2004 cohort. A summary description of the follow-up rates and the number of participants in whom physical activity was assessed by wrist accelerometry is displayed in Table 1. Individuals participating in the follow-up measurements do not differ according to SES at birth from those lost to follow-up.^6–8^

**Table 1.** Description of the most recent follow-up visits to each of the three Pelotas (Brazil) birth cohorts

Pelotas birth cohort	Mean (SD) age at follow-up	Cohort members (N)	Participants at the most recent follow-up (%)	Participants with PA measurements (%)	Participants with valid PA data (%)^a^	Participants with at least 2 valid days (%)^b^
2004	6.7 (0.19)	4137	3816 (90.2)	3331 (93.7)^c^	2642 (69.3)^c^	2636 (69.1)^c^
1993	18.4 (0.32)	5249	4106 (81.4)	3822 (93.1)	3629 (88.4)	3622 (88.2)
1982	30.2 (0.35)	5914	3701 (68.1)	2876 (77.7)	2731 (73.8)	2716 (73.4)
Total	-	15 300	11 623 (76.0)	10 029 (86.3)	9002 (77.4)	8974 (77.2)

PA, physical activity. The proportions with valid PA data and with 2+ valid days are expressed as percentage sof participants attending the most recent follow-up visit.

^a^At least one full 24-h cycle of measurement and calibration error <0.02.

^b^At least 2 valid days of measurement according to the protocol.

^c^The 165 participants who were interviewed at home were not eligible for accelerometry.

### Accelerometry

The GENEActiv accelerometer is waterproof and measures acceleration in three axes (x, y, z) within a ±8 *g* dynamic range with a sampling frequency set at 85.7 Hz. Data are stored directly as sampled from the MEMS chip (unfiltered) and expressed in units milli-*g* (1000 m*g* = 1 *g* = 9.81 m/s^2^). In a recent validation study, a strong agreement for the vector magnitude of wrist acceleration (m*g*) was observed between GENEActiv and Actigraph accelerometers as used by the NHANES survey^10^ (intra-class correlation >0.95) and the output from both monitors explained more than 70% of the variance in measured oxygen consumption across six different activities of varying intensity.^11^

**Figure 1.**
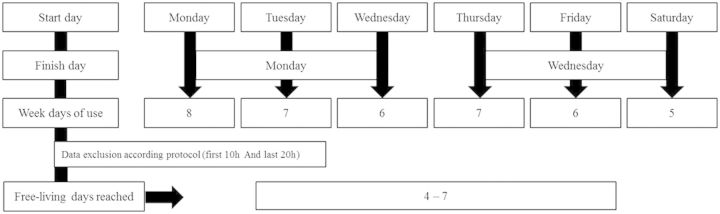
Flow diagram from the accelerometry protocol – 1982, 1993 and 2004 Pelotas birth cohorts.

For practical reasons and to increase compliance, physical activity was assessed using a 24-h protocol for 4–7 free-living days including at least one weekend day, in all participants. The total amount of monitored days varied according to the day of the clinical visit. Participants who visited the clinic on Mondays, Tuesdays or Wednesdays were monitored until the following Monday, whereas those who visited the clinic on Thursdays, Fridays or Saturdays, were monitored until the following Wednesday (Figure 1). Following the free-living measurements, accelerometers were collected by the research team at the participant’s home or workplace. Participants who were disabled or living in other cities were excluded from the measurements, as well as those who were unable to wear the monitors on their wrist during work. Women who were pregnant during the visit were contacted after delivery and invited to wear the accelerometer.

A total of 514 children from the 2004 birth cohort were assessed at the beginning of the follow-up with the non-commercial GENEA accelerometer,^12^ the production of which was terminated unexpectedly. This monitor was replaced with the commercial GENEActiv which was then used throughout the study. Due to uncertainties about consistency between GENEActiv and GENEA output, all individuals who were monitored using the GENEA device were excluded from the present analyses. As a result, only 2636 (69.1%) of the eligible participants from the 2004 cohort were included (Table 1).

### Accelerometer data processing and analysis

Accelerometers were set up and downloaded in the GENEActiv software. Accelerometer data in binary format were analysed with R-package GGIR [http:/cran.r-project.org].^13^ Summary measures used for present analyses are the average magnitude of wrist acceleration (overall volume), the distribution of time spent across acceleration levels, and estimated time spent in 5- and 10-min bouts of MVPA. The detailed signal processing scheme included the following steps: verification of sensor calibration error using local gravity as a reference;^14^ detection of sustained abnormally high values; non-wear detection; calculation of the vector magnitude of activity-related acceleration using the Euclidian Norm minus 1 *g* (ENMO:$\;\sqrt{x^{2}+y^{2}+z^{2}}-1\text{g}$) with any negative values rounded up to zero; exclusion of the first 10 and last 20 h of the measurement; and imputation of invalid data segments by the average of similar time-of-day data points on different days of the measurement.^13^ The first 10 h and the last 20 h of data in each raw accelerometer file were excluded because these were the maximum periods between initialization and attachment, and between collection of the monitors and download, respectively. Files were considered appropriate for analyses if post-calibration error was lower than 0.02 *g* and valid data were present for every 15-min period in a 24-h cycle (even when scattered over multiple days). Supplementary Figure 1 (available as Supplementary data at *IJE* online) shows an example of the acceleration over time (15-min averages) of one participant, with a 10- and a 20-h section excluded at the beginning and end of the measurement (hatched area) and the periods detected as accelerometer non-wear time (grey area).

Non-wear time was inferred from the standard deviation and value range of each accelerometer axis in 60-min windows with 15-min moving increments. A time window was classified as non-wear time if, for at least two out of the three axes, the standard deviation was less than 13 m*g* and the value range was less than 50 m*g*. By using a 60-min time window, the method aims to detect periods of monitor non-wear time lasting for more than 1 h, which are the periods that would most impact on summary measures. Further, using this time window ensured that short periods of inactivity or even sleep were not confused with non-wear time.^13^

The summary measure ENMO as described above was used as an indicator of average magnitude of dynamic wrist acceleration over the measurement period. The distribution in time spent across acceleration levels was investigated in 40-m*g* resolution. We also present mean minutes per day spent in MVPA using an intensity threshold of 100 m*g* based on 5-s epoch data and two different minimum bout durations (5 and 10 min). The intensity threshold is based on a recent methodological study in children and adults wearing a GENEActiv accelerometer on their wrist while performing standardized activity types. In this study, 100 m*g* was found to be within the range of acceleration values corresponding to walking in children and adults, a round number so as not to give the impression of over-precision, and close to the estimate for 3 metabolic equivalents of task (MET) for adults.^11^ Bouts of MVPA are identified as all 5- or 10-min time windows that start with a 5-s epoch value equal to or higher than 100 m*g* and for which 80% of subsequent5-s epoch values are equal to or higher than the 100-m*g* threshold.

### Stratifying variables

Stratifying variables to describe physical activity were: (i) sex (male/female); (ii) birth cohort (1982, 1993 and 2004); (iii) weight status (underweight, normal-weight, overweight and obese) based on the World Health Organization (WHO) classification of BMI for adults^15^ (1982 cohort) and according to the age and sex Z-scores for BMI for children and adolescents (1993 and 2004 cohorts);^16^ and (iv) SES generated by a standardized socioeconomic questionnaire, including questions on household assets, the presence of a maid, and education level (categorized in quintiles based on a principal component analysis).^17^

### Statistics

Descriptive analyses were performed using Stata 12.0 based on valid data from all participants who provided at least 2 days of measurement according to the protocol. One-way analysis of variance (ANOVA) or non-parametric Kruskal-Wallis testing were used to compare acceleration mean differences across age groups (different birth cohorts), weight status and SES. The Wilcoxon test was used to compare means between males and females. Multiple linear regressions using multiplicative terms were carried out to examine potential interactions between variables. Additionally, multivariate analysis of variance through Wilks’ lambda test was used to compare acceleration distribution differences according to birth cohort and sex. Statistical significance was set at 5%, and 95% confidence intervals are provided.

## Results

The proportion of males in the samples varied from 48.3% in the 1982 cohort to 51.5% in the 2004 cohort. The prevalence of obesity was 24% in the oldest cohort, 9.7% in the 1993 cohort and 16.9% in the youngest cohort. In total, 8974 individuals provided valid data on objectively measured physical activity including at least 2 days of measurement (Table 1). Overall, participants provided an average of 5.0 [standard deviation (SD) = 1.0 and interquartile range 4.6–5.7) days of measurement following exclusion of the first 10 and last 20 h for each individual. No differences in monitor wear time were found in terms of sex, SES or weight status (means varied only between 4.7 and 5.2 days). The mean total duration of non-wear time was 4.6  (SD = 13.0) hours across the entire measurement period, representing 3.6% of the total measurement duration. Furthermore, 61% of the participants had zero hours of non-wear across the total measurement period. Although non-wear time was not influenced by sex or SES, it was higher in the 1982 and 1993 birth cohorts [4.0% (SD = 9.8) and 5.2% (SD = 12.1), respectively] compared with the 2004 birth cohort [1.1% (SD = 5.2)].

**Figure 2.**
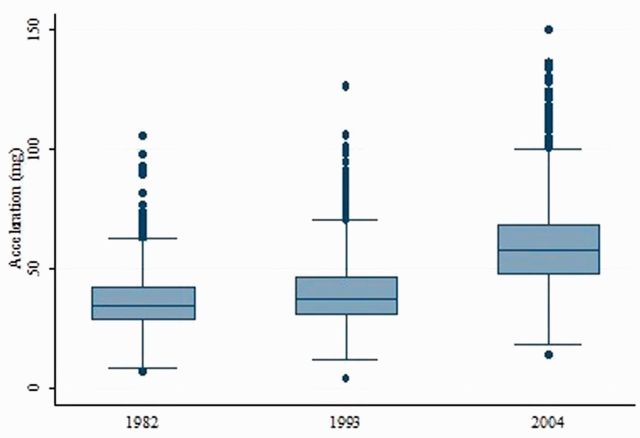
Acceleration in the Pelotas (Brazil) birth cohorts.

Figure 2 shows the box-and-whisker plot of average wrist acceleration magnitude used as an indicator of overall physical activity in the three cohorts. The sample means (95% CI) in the 1982, 1993 and 2004 birth cohorts were 35.2 m*g* (34.8; 35.6), 38.7 m*g* (38.3; 39.2), and 60.0 m*g* (59.3; 60.7), respectively. An inverse linear association between mean acceleration and age of the cohorts was observed.

Males had higher overall levels of physical activity than females across the three cohorts (Table 2). However, an age-by-sex interaction was observed; there were smaller acceleration differences across age groups in females, compared with males. Indeed, the mean acceleration in the two older cohorts (1982 and 1993 birth cohorts) in females was similar (32.7 and 34.5 m*g*, respectively). Among males, mean acceleration was 37.8 m*g* (95% CI 37.1; 38.4) and 43.0 m*g* (95% CI 42.3; 43.7) in the two older cohorts, respectively. In both sexes, mean acceleration in the youngest cohort (2004) was higher (56.0% higher among males and 60.6% among females) compared with the two older cohorts (1982 and 1993).

**Table 2.** Acceleration means and 95% confidence intervals in the three Pelotas birth cohorts

Cohort (mean age)	Males				Females				*P*^b^
	N	Acc mean (m*g*)	95% CI	*P*^a^	N	Acc mean (m*g*)	95% CI	*P*^a^	
				<0.001				<0.001	
1982 cohort (30 y)	1310	37.8	37.1–38.4		1403	32.7	32.2–33.2		<0.001
1993 cohort (18 y)	1770	43.0	42.3–43.7		1838	34.5	34.1–35.0		<0.001
2004 cohort (7 y)	1353	64.6	63.6–65.7		1273	55.1	54.3–55.9		<0.001

Acc, acceleration.

^a^Kruskal-Wallis test comparing differences across cohorts.

^b^Wilcoxon test comparing differences by sex.

Overall physical activity expressed as mean acceleration, stratified by cohort, sex and SES status, are shown in Table 3. For all sex and age groups, there was an inverse linear association between acceleration and SES. Higher accelerations were observed in the poorer SES quintiles. Table 4 presents mean acceleration by weight status categories, stratified by sex and birth cohort. There was no difference in overall physical activity by weight status among females in the 1982 and 1993 cohorts, whereas normal-weight females in the 2004 cohort were more active than those categorized as underweight or obese. Among males there were differences in the means of acceleration in dissimilar groups of weight status across the three cohorts. Normal-weight individuals had consistently higher acceleration means, especially compared with those categorized as underweight or obese.

**Table 3.** Acceleration mean and 95% confidence interval by socioeconomic status quintiles in the three Pelotas (Brazil) birth cohorts, for males and for females

Socioeconomic quintiles	1982 cohort (30 y)				1993 cohort (18 y)				2004 cohort (7 y)			
	N	Acc mean (m*g*)	95% CI	*P*^a^	N	Acc mean (m*g*)	95% CI	*P*^a^	N	Acc mean (m*g*)	95% CI	*P*^a^
	Males											
				<0.001				<0.001				<0.001
Q1 (poorest)	272	39.4	38.0–40.9		284	47.8	46.1–49.5		134	68.3	65.4–71.1	
Q2	264	38.7	37.4–40.1		355	47.4	45.8–49.1		195	67.7	65.0–70.5	
Q3	330	39.5	38.3–40.7		356	43.6	42.2–45.0		297	64.6	62.6–66.7	
Q4	110	37.3	35.3–39.4		394	41.7	40.4–43.1		287	62.3	61.0–65.6	
Q5 (wealthiest)	240	35.3	33.9–37.7		370	39.1	37.8–40.3		405	61.0	59.4–62.7	
	Females											
				<0.001				<0.001				<0.001
Q1 (poorest)	363	34.5	33.6–35.5		436	37.7	36.8–38.7		125	59.2	56.5–61.9	
Q2	274	34.7	33.6–35.8		354	36.0	35.1–36.9		204	56.8	55.0–58.7	
Q3	336	33.3	32.3–34.2		366	34.8	33.9–35.9		297	55.8	54.2–57.4	
Q4	127	31.8	30.4–33.1		344	34.4	33.3–35.5		225	52.9	51.2–54.7	
Q5 (wealthiest)	217	30.2	29.2–31.3		331	32.1	31.6–33.0		386	52.3	51.2–53.5	

^a^*P*-values represent the result of Kruskal-Wallis tests for comparing acc means (m*g*) across socioeconomic quintiles in each cohort.

**Table 4.** Acceleration mean and 95% confidence interval by weight status in the three Pelotas (Brazil) birth cohorts, for males and females

Weight status	1982 cohort (30 y)				1993 cohort (18 y)				2004 cohort (7 y)			
	N	Acc mean (m*g*)	95% CI	*P*^a^	N	Acc mean (m*g*)	95% CI	*P*^a^	N	Acc mean (m*g*)	95% CI	*P*^a^
	Males											
				0.03				<0.001				<0.001
Underweight	19	36.6	30.8–42.3		24	35.4	30.6–40.3		15	64.2	51.9–76.5	
Normal	473	39.3	38.2–40.4		1293	45.0	44.2–45.8		806	66.5	65.2–67.7	
Overweight	503	38.5	37.6–39.5		291	41.4	39.9–42.9		230	61.5	59.3–63.7	
Obesity	292	36.8	35.5–38.0		151	39.5	37.6–41.4		216	57.5	55.7–59.4	
	Females											
				0.07				0.40				<0.001
Underweight	36	34.3	31.3–37.4		24	32.9	28.7–37.0		9	55.1	41.3–69.0	
Normal	590	33.6	32.8–34.3		1276	35.1	34.5–35.6		768	56.1	55.1–57.1	
Overweight	406	33.6	32.7–34.4		334	35.9	34.9–37.0		224	54.0	52.5–55.5	
Obesity	338	32.2	31.3–33.0		197	35.2	33.9–36.5		200	51.5	49.7–53.3	

^a^*P*-values represent the result of Kruskal-Wallis tests for comparing acc means (m*g*) across weight status categories in each cohort.

**Figure 3.**
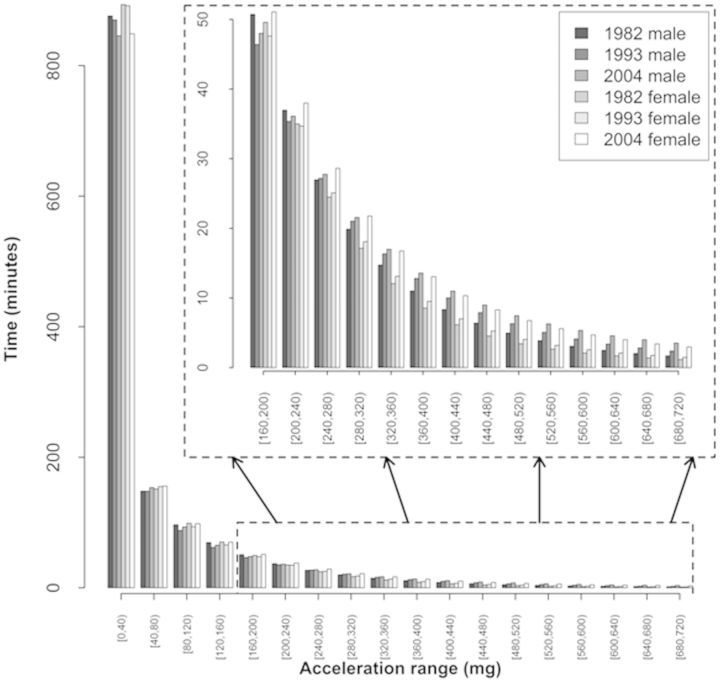
Acceleration distribution of time spent in 40 m*g* categories (intensity) in the three Pelotas (Brazil) birth cohorts stratified by sex. *The distributions of accelerometry across cohorts were statistically significant (p = 0.000) according to the Wilks' lambda test.

Figure 3 and Supplementary Table 1 (available as Supplementary data at *IJE* online) show the acceleration distribution of time spent in 40-m*g* categories (intensity) for the three birth cohorts stratified by sex. The distributions were different across birth cohorts in both sexes; more time per day was spent at lower activity intensities in the oldest cohort, compared with the younger ones.

Data on time spent in MVPA using the two different bout criteria (5 and 10 min) are displayed in Table 5. Mean MVPA in 10-min bouts were 26 (95% CI 25; 27), 43 (95% CI 42; 44) and 45 (95% CI 43; 46) min per day in the 1982, 1993 and 2004 cohorts, respectively, with corresponding 29.6% (95% CI 27.9; 31.5), 52.8% (95% CI 51.2; 54.5) and 59.6% (95% CI 57.8; 61.5) of participants accumulating at least 30 min of MVPA per day. Mean MVPA in the oldest cohort was 50% higher when using a 5-min bout criterion; it was 35% higher in the 1993 cohort and 40% higher in the youngest cohort. The associations between weight status and SES groups and time spent in MVPA were similar to those observed for average acceleration.

**Table 5.** Mean time spent per day in moderate to vigorous physical activity (MVPA) and 95% confidence interval with two different bout lengths in the three Pelotas (Brazil) birth cohorts^a^

Variables	1982 cohort (aged 30 y)			1993 cohort (aged 18 y)			2004 cohort (aged 7 y)		
	N	5-min bout	10-min bout	N	5-min bout	10-min bout	N	5-min bout	10-min bout
Gender									
Male	1309	47 (44–49)	32 (30–34)	1762	75 (72–77)	58 (55–60)	1341	74 (72–76)	55 (53–57)
Female	1402	31 (29–32)	19 (18–20)	1829	41 (40–43)	29 (27–30)	1261	50 (49–52)	33 (32–35)
Socioeconomic quintiles									
Q1 (poorest)	635	46 (43–49)	31 (29–34)	720	65 (61–68)	48 (45–51)	256	74 (69–79)	53 (48–57)
Q2	539	42 (39–45)	28 (26–30)	706	67 (63–71)	50 (47–54)	394	68 (63–72)	48 (44–51)
Q3	666	37 (34–40)	24 (21–26)	716	58 (55–61)	43 (41–46)	589	64 (60–67)	45 (42–48)
Q4	237	32 (28–36)	21 (17–25)	736	55 (52–59)	42 (39–45)	511	62 (59–65)	45 (42–48)
Q5 (wealthiest)	455	26 (23–28)	17 (15–19)	695	43 (40–46)	32 (29–34)	781	56 (53–58)	37 (37–42)
Weight status									
Underweight	55	38 (30–47)	25 (19–32)	48	47 (37–58)	37 (27–47)	24	69 (47–92)	53 (33–73)
Normal	1063	41 (39–43)	28 (26–30)	2556	60 (59–62)	45 (44–47)	1556	67 (65–69)	48 (46–50)
Overweight	909	40 (38–43)	27 (25–29)	622	53 (50–56)	38 (35–41)	452	59 (55–62)	42 (39–45)
Obesity	629	32 (29–34)	20 (18–22)	347	48 (44–52)	34 (31–38)	413	52 (49–56)	36 (33–39)

^a^Non-parametric Wilcoxon tests were used to compare mean MVPA between males and females. Non-parametric Kruskal-Wallis tests were used to compare mean MVPA by socioeconomic and weight status categories. All *P-*values were <0.001, and are therefore not presented in the table.

## Discussion

The current study presents data on physical activity by raw triaxial wrist accelerometry in almost 9000 participants, resulting in 46 176 person-days of measurement from three birth cohorts in Pelotas, Brazil. Mean MVPA time in 10-min bouts was below 1 h per day in all cohorts, and even below 30 min per day in the 1982 cohort. Differences were found across cohorts, sex, SES and weight status groups.

Few if any previous studies have provided population estimates of physical activity obtained by wrist-mounted raw accelerometers. Thus, a direct comparison between our results with others is difficult. However, the differences between age groups are in line with other studies.^18^ The main difference in terms of overall physical activity (expressed in average acceleration magnitude) was observed when comparing participants from the 1993 (43.0 m*g* in males and 34.5 m*g* in females) and 2004 (64.6 m*g* in males and 55.1 m*g* in females) birth cohorts. The pronounced negative difference in overall physical activity between childhood and older adolescence corroborates previous findings using hip accelerometry^19^^,^^20^ and suggests that interventions targeted at increasing physical activity in teenagers is highly relevant in low- and middle-income countries.

Interestingly, when considering MVPA bouts instead of overall acceleration, the largest difference was between the 1982 (26 min/day) and the 1993 (43 min/day) cohorts. These findings indicate differences in how physical activity is patterned in children and adolescents as compared with adults. Children’s movements typically include short periods of high intensity physical activity, and therefore tend to be underestimated by the use of long bout durations. The marked difference between time spent in MVPA bouts between adults and adolescents is likely also explained by differences in movement patterns between groups. Adolescents are more frequently involved in organized sport activities which usually include a substantial amount of time in MVPA of at least 5-min duration. It is also plausible that adolescents are more likely to be physically active at least at moderate intensity for longer periods of time in transport-related activity compared with adults, simply due to less access to cars.

An inverse association between age of the cohorts and physical activity was observed in both sexes, but the differences tended to be attenuated over time (age). The absolute difference between males and females in mean acceleration was 9.5 m*g* in the 2004 cohort, 8.5 m*g* in the 1993 and 5.1 m*g* in the 1982 cohort. The likely explanation for the age differences being more marked among boys is the fact that overall physical activity values are much lower in younger girls, leaving less room for age-related decline.

A systematic review reported a positive association between physical activity and SES in low- and middle-income countries.^17^ In contrast, we observed an inverse association between activity (both mean acceleration and MVPA) and SES in all groups, similar to a previous study in children aged 4–11 years from the same city also using accelerometry.^4^ These findings are likely explained by the fact that several of the studies summarized in the review focused solely on leisure-time physical activity,^18^ which may be more frequent among high-SES people, but one cannot exclude socially patterned reporting bias as an alternative explanation. These discrepant findings highlight the need for implementing objective methods when examining correlates and determinants of physical activity, particularly in low- and middle-income countries where a significant proportion of total physical activity takes place in domains other than leisure time.^21^ In our cohorts, for example, self-reported data indicate that transport-related physical activity is much more frequent among low-SES as compared with high-SES participants.^22^

Male participants classified as normal-weight were more active than the other weight status groups. In contrast, this association was not consistent among females. The association between physical activity and weight status may be differentially confounded by socioeconomic position although the observed magnitude of associations between physical activity and weight status were almost unchanged after adjustment for SES status (data not shown). In the Pelotas cohorts it has been shown that low-SES females are more likely to be overweight than those of higher SES status. In contrast, a higher prevalence of overweight and obesity is observed in those from higher SES status in males.^23^^,^^24^ Finally, caution is warranted when interpreting cross-sectional associations between physical activity and weight status due to the possibility of reversed causality.

Participants from the oldest cohort (30 y) spent, on average, less than 30 min per day in MVPA, whereas participants from the two other cohorts spent around 45 min per day in such activities. Taking into account that WHO guidelines recommend daily 30 min of MVPA for adults and 60 min of MVPA for children and adolescents, the average values reported here are below the threshold in all cases. However, if we simply change the minimum bout duration to 5 instead of 10 min, these figures rise considerably, reaching almost 40 MVPA min per day in those aged 30 years and around 1 h per day in those aged 18 or 7 years. These findings highlight how simple changes in analysis decisions can lead to completely different conclusions in terms of mean MVPA and proportion of the population reaching physical activity guidelines. Standardization efforts are needed so that studies using objectively measured physical activity can be compared across populations, and further aetiological work is needed to establish the possible health benefits of varying bout durations.

The evaluation on non-wear periods suggested high compliance with wearing the monitors. A wrist protocol appears more acceptable compared with the traditional waist attachment,^25^ and may therefore provide a less biased estimate of daily physical activity. However, age differences should be taken into account; non-wear was higher in the older cohort, most likely because older individuals may have removed the monitor during social activities. Even so, average percentage wear time was still 94.8% and 96.1% of the total period in the 1993 and 1982 cohorts, respectively.

An important feature of the current study was the collection of raw triaxial accelerometry signals, allowing full control of all stages of data processing and a higher comparability between accelerometer brands. That said, raw accelerometry data from different brands may still be subjective to analog filtering, e.g. including an anti-aliasing filter which is not present in the GENEActiv as used in the current study. Applying different filtering regimes to acceleration signals will impact on results to a varying degree, as demonstrated in our earlier work.^13^^,^^26^

Some limitations should be taken into account. The decisions to delete the first 10 h and the last 20 h may have caused the omission of real behavioural data from some of our participants. However, our conservative decision to delete this information ensured that the data used for analysis only included acceleration corresponding to periods of time when the activity monitor was with the participant and not being transported; the motion during delivery and collection of the accelerometer to and from the participant would confuse the non-wear detection. An additional limitation is that we did not provide any estimates of waking sedentary time as no valid sleep detection algorithms are currently available. Nonetheless, this is the largest study to date on objective physical activity assessment in low- and middle-income countries, with raw accelerometer data collected in 8974 people and allowing a description of differences in physical activity across socio-demographic groups and weight status. The high response rate for all three birth cohorts, as well as the high percentage of individuals of each birth cohort that provided valid data, are strengths of this study.

In conclusion, our results suggest that Brazilian males are more active than females and physical activity is higher in children and lowest in adults. Further, physical activity was lower in higher SES groups in all cohorts. Physical activity was also higher in normal-weight individuals as compared to underweight, overweight and obese participants. Future studies using objective assessment of physical activity are needed to establish time trends in physical activity in low- and middle-income countries undergoing rapid epidemiological transition.

## Supplementary Data

Supplementary data are available at *IJE* online.

## Funding

This analysis was supported by the Wellcome Trust (WT086974MA). Earlier phases of the 1982, 1993 and 2004 cohort studies were funded by: the International Development Research Center (Canada); the World Health Organization (Department of Child and Adolescent Health and Development, and Human Reproduction Programme); the Overseas Development Administration (currently Department for International Development) (UK); the European Union; the United Nations Development Fund for Women; the National Program for Centers of Excellence (PRONEX/CNPq/FAPERGS) (Brazil); the Pastorate of the Child (Brazil); the National Council for Scientific and Technological Development (CNPq), (Brazil); and the Ministry of Health (Brazil). P.C.H is funded by the Wellcome Trust through a New Investigator Award; S.B. and U.E. are partially funded from the UK Medical Research Council (MC_UU_12015/3); and I.da S. is funded by the Brazilian Federal Agency for the Support and Evaluation of Graduate Education (CAPES) through a scholarship.

## Supplementary Material

Supplementary Data
