# Supplementary material for: Physical activity levels in three Brazilian birth cohorts as assessed with raw triaxial wrist accelerometry
Source: Int J Epidemiol. 2014 Oct 30;43(6):1959–68. doi: 10.1093/ije/dyu203 (PMC4276065; doi:10.1093/ije/dyu203)
Supplement: Supplementary Data [file supp_43_6_1959__index.html]

Physical activity levels in three Brazilian birth cohorts as assessed with raw triaxial wrist accelerometry — Physical activity levels in three Brazilian birth cohorts as assessed with raw triaxial wrist accelerometry — Supplementary Data 

# Physical activity levels in three Brazilian birth cohorts as assessed with raw triaxial wrist accelerometry

## Supplementary Data

files

**Files in this Data Supplement:**

- Supplementary Data - png file
- Supplementary Data - doc file
