# Supplementary figures and images for: Physical activity levels in three Brazilian birth cohorts as assessed with raw triaxial wrist accelerometry
Source: Int J Epidemiol. 2014 Oct 30;43(6):1959–68. doi: 10.1093/ije/dyu203 (PMC4276065; doi:10.1093/ije/dyu203)

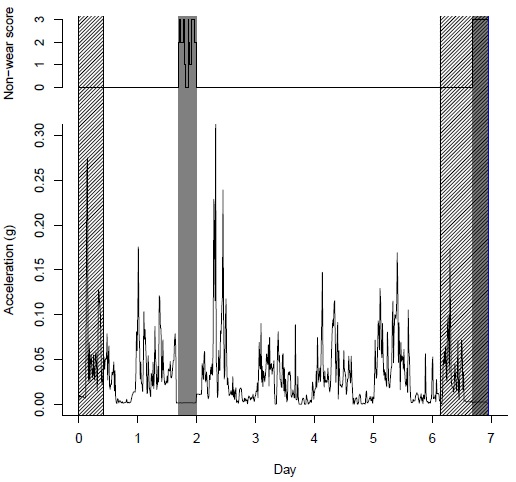

Supplement: Supplementary Data [file supp_dyu203_ije-2013-12-1259-File005.png]
