# Supplementary material for: Physical activity levels in three Brazilian birth cohorts as assessed with raw triaxial wrist accelerometry
Source: Int J Epidemiol. 2014 Oct 30;43(6):1959–68. doi: 10.1093/ije/dyu203 (PMC4276065; doi:10.1093/ije/dyu203)
Supplement: Supplementary Data [file supp_dyu203_ije-2013-12-1259-File006.doc]

Supplementary Table 1. Average of minutes per day spent in 40 mg categories by birth cohort .

| **Birth Cohort** | **Minutes per day spent in Mg categories** | | | | | | | | | | | | | | | | | | | | **P-value*** |
| --- | --- | --- | --- | --- | --- | --- | --- | --- | --- | --- | --- | --- | --- | --- | --- | --- | --- | --- | --- | --- | --- |
|  | [0,40) | [40, 80) | [80, 120) | [120,160) | [160,200) | [200,240) | [240,280) | [280,320) | [320,360) | [360,400) | [400,440) | [440,480) | [480,520) | [520,560) | [560,600) | [600,640) | [640,680) | [680,720) | [720,760) | [760,800) |  |
| **Males** | | | | | | | | | | | | | | | | | | | | | <0.001 |
| **1982** | 1024.2 | 166.0 | 87.71 | 46.86 | 25.75 | 14.75 | 8.86 | 5.57 | 3.72 | 2.57 | 1.86 | 1.36 | 1.03 | 0.78 | 0.62 | 0.48 | 0.39 | 0.31 | 0.26 | 0.22 |  |
| **SD** | 212.9 | 52.3 | 33.9 | 21.2 | 13.6 | 8.8 | 5.7 | 3.9 | 2.7 | 2.0 | 1.5 | 1.2 | 1.0 | 0.8 | 0.7 | 0.6 | 0.5 | 0.4 | 0.4 | 0.4 |  |
| **1993** | 1018.0 | 149.9 | 81.76 | 48.22 | 29.15 | 17.93 | 11.39 | 7.51 | 5.21 | 3.76 | 2.79 | 2.09 | 1.62 | 1.27 | 1.00 | 0.82 | 0.67 | 0.56 | 0.47 | 0.41 |  |
| **SD** | 227.6 | 49.8 | 32.5 | 22.1 | 14.7 | 9.9 | 6.8 | 4.9 | 3.8 | 3.0 | 2.5 | 1.8 | 1.5 | 1.2 | 1.0 | 0.8 | 0.7 | 0.6 | 0.5 | 0.5 |  |
| **2004** | 999.2 | 158.6 | 84.12 | 49.35 | 30.59 | 20.00 | 13.74 | 9.94 | 7.54 | 5.92 | 4.76 | 3.96 | 3.33 | 2.83 | 2.44 | 2.14 | 1.87 | 1.65 | 1.48 | 1.31 |  |
| **SD** | 141.5 | 31.9 | 19.2 | 13.2 | 9.2 | 6.6 | 4.9 | 3.7 | 2.9 | 2.3 | 1.9 | 1.7 | 1.4 | 1.3 | 1.1 | 1.0 | 0.9 | 0.8 | 0.7 | 0.7 |  |
| **Females** | | | | | | | | | | | | | | | | | | | | | <0.001 |
| **1982** | 1044.7 | 169.2 | 84.60 | 41.64 | 20.61 | 10.69 | 6.08 | 3.78 | 2.53 | 1.76 | 1.26 | 0.93 | 0.70 | 0.53 | 0.41 | 0.32 | 0.24 | 0.19 | 0.15 | 0.12 |  |
| **SD** | 217.8 | 53.3 | 31.2 | 18.4 | 10.6 | 6.3 | 3.9 | 2.6 | 1.9 | 1.5 | 1.2 | 1.0 | 0.8 | 0.7 | 0.5 | 0.5 | 0.4 | 0.3 | 0.3 | 0.3 |  |
| **1993** | 1046.5 | 159.3 | 82.30 | 43.18 | 22.63 | 12.27 | 7.25 | 4.66 | 3.21 | 2.28 | 1.67 | 1.26 | 0.96 | 0.74 | 0.57 | 0.45 | 0.35 | 0.28 | 0.22 | 0.17 |  |
| **SD** | 217.8 | 50.8 | 31.1 | 18.5 | 10.8 | 6.5 | 4.3 | 2.9 | 2.2 | 1.7 | 1.4 | 1.1 | 0.9 | 0.8 | 0.7 | 0.6 | 0.5 | 0.4 | 0.3 | 0.3 |  |
| **2004** | 1004.4 | 168.1 | 89.06 | 50.38 | 29.80 | 18.63 | 12.33 | 8.67 | 6.36 | 4.88 | 3.86 | 3.14 | 2.61 | 2.18 | 1.86 | 1.59 | 1.39 | 1.21 | 1.07 | 0.94 |  |
| **SD** | 135.1 | 30.4 | 19.8 | 13.4 | 9.1 | 6.3 | 4.4 | 3.3 | 2.5 | 1.9 | 1.6 | 1.3 | 1.1 | 0.9 | 0.8 | 0.7 | 0.7 | 0.6 | 0.5 | 0.5 |  |

The symbol [0,40) denotes greater than and equal to 0 and smaller than 40 (according to ISO 31-11).

SD – Standard deviation

* Wilk’s lambda test
